# Bidirectional Behavioral Selection in Mice: A Novel Pre-clinical Approach to Examining Compulsivity

**DOI:** 10.3389/fpsyt.2021.716619

**Published:** 2021-09-08

**Authors:** Swarup Mitra, Abel Bult-Ito

**Affiliations:** ^1^Department of Pharmacology and Toxicology, State University of New York at Buffalo, Buffalo, NY, United States; ^2^Department of Biology and Wildlife, University of Alaska Fairbanks, Fairbanks, AK, United States; ^3^OCRD Biomed LLC, Fairbanks, AK, United States

**Keywords:** OCD, artificial selection, spontaneous compulsive-like phenotype, behavioral heterogeneity, face-predictive-construct validity, desformylflustrabromine, OCRD

## Abstract

Obsessive-compulsive disorder (OCD) and related disorders (OCRD) is one of the most prevalent neuropsychiatric disorders with no definitive etiology. The pathophysiological attributes of OCD are driven by a multitude of factors that involve polygenic mechanisms, gender, neurochemistry, physiological status, environmental exposures and complex interactions among these factors. Such complex intertwining of contributing factors imparts clinical heterogeneity to the disorder making it challenging for therapeutic intervention. Mouse strains selected for excessive levels of nest- building behavior exhibit a spontaneous, stable and predictable compulsive-like behavioral phenotype. These compulsive-like mice exhibit heterogeneity in expression of compulsive-like and other adjunct behaviors that might serve as a valuable animal equivalent for examining the interactions of genetics, sex and environmental factors in influencing the pathophysiology of OCD. The current review summarizes the existing findings on the compulsive-like mice that bolster their face, construct and predictive validity for studying various dimensions of compulsive and associated behaviors often reported in clinical OCD and OCRD.

## Introduction

The prevalence of obsessive-compulsive disorder (OCD) ranges from 1 to 3% (1, 2) including a lifetime prevalence of 1.6% (3) to 2.3% (4) and a 12-month prevalence of 1.2% in the United States (US) (4), and a point prevalence of 3.3% in India (5) and 1.7% in Greece (6). It was first described as a psychiatric condition about 100 years ago (7, 8). The World Health Organization has indicated OCD to be the leading global cause of morbidity (9) that has however declined in the recent times due to refinements in the understanding of the prevalence of the condition. Previously OCD has been considered among the top 20 causes of years of life spent with disability for patients between 15 and 44 years of age (10). In 1990, costs related to OCD in the US were estimated to be $8.4 billion (11). Costs include medical care and lost productivity related to functional disabilities. OCD has a large impact on quality of life and functional disabilities including impairments in mental health, social and household functioning, work outcomes and physical health (12–19). The average time from OCD onset to initial treatment is over 7 years (20–22) adding to the patient's burden of decreased quality of life during a significant period of their lives. This is mainly because patients are inclined to conceal their symptoms fearing they will appear abnormal and seek medical attention only when other co-morbid conditions, such as anxiety and depression, exacerbate the disorder (23, 24). Ineffective identification of specific symptoms is also one of the reasons for delayed diagnosis (25).

OCD patients suffer from persistent obsessive thoughts and compulsive repetitive behaviors to alleviate uncomfortable feelings of anxiety and distress (23, 26–30). Compulsions are typically meant to neutralize the obsessive feelings (31), but provide only transient relief leading to reinforcement of the behaviors and continuation of the obsession-compulsion cycle (29). Obsessions can be thematic, such as fear of contamination, pathological doubt, or need for symmetry/order. Compulsive behaviors involve washing, seeking, counting, sorting, checking and searching (23, 26–30). Some patients experience obsessions-only symptoms without any compulsive ritualistic behaviors. An example is that of fear of causing harm to others or self (29). Many studies use the Yale-Brown Obsessive-Compulsive Scale (Y-BOCS scale), an 10-item clinically administered scale (29), to clinically characterize and identify the occurrence and types of obsessive-compulsive symptoms (32). Further enhancements to this scale involve the Dimensional Yale-Brown Obsessive-Compulsive Scale (DY-BOCS) which categorizes OCD into 6 distinct dimensions (33). They are (i) aggression/checking, (ii) sexual/moral/religious, (iii) symmetry/ordering/counting, (iv) contamination/washing, (v) hoarding, and (vi) others (29, 33, 34). In addition, researchers have divided OCD into subgroups based on familiality, gender, age of onset and comorbid patterns (35–38). The specific OCD dimensions vary from study to study (4, 29, 36, 39–41), which shows no consensus in the field. This heterogeneity in OCD symptoms complicates identification of candidate genes (29) and the choice of initial pharmacotherapy, which may explain why 40–60% of OCD patients do not respond to initial selective serotonin reuptake inhibitor (SSRI) treatment (42, 43), requiring repeated treatments with different drugs until a response is observed (44).

The age of onset of OCD ranges from early in childhood to adulthood (29). Onset after 30 years of age is highly unusual (4, 44). Approximately 30–50% of the patients have onset of symptoms before 10 years of age indicating neurodevelopmental aspects to the disorder (45–47). Interestingly, in childhood onset OCD, males are more commonly affected than females with a ratio of ~2:1–3:1 (males: females). This ratio however shifts to 1:1.4 among patients with onset during or after puberty (48). Additionally, there is a significant overlap in symptom dimensions in patient subgroups between early and late onset cohorts. Though genetic linkage, candidate gene and genome wide association studies in humans (49–54) have indicated several genetic associations, the precise causative factors remain elusive (31). This could be attributed to the heterogeneous nature of the disorder which in turn is influenced by several factors such as sex differences, environmental exposures, genetic variations, neurotransmitter systems and physiological status.

The Diagnostic and Statistical Manual of Mental Disorders 5 (DSM-5) now classifies OCD within obsessive-compulsive and related disorders (OCRD) (55). OCRD include OCD (23, 26–30, 56), excoriation disorder (excessive skin picking) (57–62), trichotillomania (excessive hair pulling) (57, 61, 63–67), onychophagy (excessive nail biting) (57, 61, 68), body dysmorphic disorder (excessive/obsessive thinking about one or more perceived defects or flaws in one's appearance that are minor or not obvious to others) (69–76), and hoarding disorder (excessive collection of objects of limited use) (77–82). The most common comorbid disorders associated with OCD are depression and anxiety (83–86). Other less frequently observed comorbid symptoms include social phobia, Tourette's syndrome, bipolar disorder, attention deficit-hyperactivity disorder, dysthymic disorder, alcohol use disorder, eating disorders, and other OCRD (87–98). One of the challenges associated with such elaborate symptom and comorbidity features of OCD is finding a relevant animal model that enables examining the neural and behavioral underpinnings underlying the clinical aspects of the disorder.

OCD is most likely polygenic (29, 99, 100), although genes of major effect cannot be ruled out, especially for small groups of OCD patients and dimensions of OCD (101–103). Heritability estimates for OCD vary from 26 to 65%, which may depend on symptom dimension (29, 39, 104–106). Genes involved in serotonergic, dopaminergic and glutamatergic signaling pathways have been implicated with pathway-specific pharmacotherapy for OCD (107–125). Genetic linkage, candidate gene and genome-wide association studies have identified many gene candidates that may affect OCD or convey a susceptibility for development of OCD (29, 126, 127), including the glutamatergic system (99, 123, 127–136). Despite all these efforts, identifying putative genes that contribute to OCD reliably and consistently across studies has been difficult because OCD is a complex, heterogeneous and most likely polygenic trait (29, 99, 100) with several genes having small effects that are difficult to reveal at the level of individual genes (29, 126). Thus, compulsive-like behaviors expressed in single gene manipulations may not be mimicking OCD, or may be relevant for only a small percentage of OCD patients (137, 138) or identify susceptibility genes for the expression of OCD (130, 139).

While prior reviews have discussed extensively the existing animal models of OCD, we only describe these animal models briefly here and focus particularly on the spontaneous compulsive-like nest-building phenotype (140–145) and argue in favor of this model as suitable for understanding behavioral plasticity underlying expression of compulsivity typically reported in OCD and OCRD.

## Spontaneous Animal Model for OCD

Spontaneous animal models use the concept of innately occurring stereotypic behaviors, motor behaviors, or adjunctive behaviors that are induced through behavioral manipulations (146–148). Animal behaviors that are suggested to be relevant to OCD (149, 150) include excessive grooming (151, 152), acral lick (153–155) and stereotopies (156, 157) in several species such as dogs, psychogenic alopecia in cats (158–160), repetitive pacing in captive wild animals (161–163), feather picking in birds (151, 164–166), repetitive behaviors, such as running, flipping and jumping in deer mice (167, 168) and cribbing in horses (169–173), spontaneous alteration (174), compulsive checking by mice and rats (174), marble burying behavior (digging) in mice (140–145, 175) and rats (149), signal attenuation in rats (176, 177), and nest-building behavior (140–145). Most of these compulsive-like behaviors in these animals have limited utility because they are quite unpredictable, have to be induced, necessitating behavioral evaluation of an entire generation, or occur sporadically. As an exception, excessive forms of grooming, nest-building and digging behaviors have shown to be predictable and have face, predictive and construct validity for understanding compulsive behaviors associated with OCD (23, 29, 99, 140–145, 178–180). Nest-building and digging behaviors in mice bidirectionally selected for excessively high and low levels of nest-building behavior remain stable across generations (181, 182).

## Induced Compulsive-Like Behaviors

Rats and mice can be induced to express compulsive-like behaviors by treatment with drugs such as dopamine (D2/D3) receptor agonist quinpirole (183–186), serotonin receptor (5HTR) agonists 5-methoxy-N,N-dimethyltryptamine (5-MeODMT; non-selective agonist), 8-hydroxy-2-(di-n-propylamino)-tetralin hydrobromide (8-OH-DPAT; 5HT-1AR agonist) (187–189) and meta-Chlorophenylpiperazine (mCPP; 5HT2CR agonist) (190, 191). The drug-induced compulsive-like behaviors provide a wealth of information on the underlying neurobiology of the disorder. For example, mCPP exacerbates OCD symptoms in clinical populations (192, 193). 5HT1BR agonist-induced OCD-like mouse model (194–197) is another model that reveals how serotonin reuptake inhibitors (SRI) attenuate OCD-like behaviors by desensitizing orbitofrontal 5HT1BRs (195). One of the limitations of these models however could be in expanding on the understanding of the onset of action (198). Behaviorally induced compulsive-like behaviors include schedule-induced polydipsia (199) and food-restriction-induced hyperactivity in rats, stress- and fear-induced marble burying in mice (149, 200) and water spray-induced grooming (201). These induced compulsive-like behaviors have varying degrees of validity (149, 200).

## Genetically Modified Mice That Express Compulsive-Like Behaviors

Mutant, gene knockout, or gene knockdown mice have been shown to express compulsive-like behaviors that are relevant to OCD. These have resulted in several interesting animals systems, which have varying degrees of validity for studying OCD (174, 199, 200, 202–205). The Hoxb8-knockout mouse was the first transgenic pre-clinical model to study OCD. Hoxb8-knockout leads to pathological grooming behavior which is triggered from aberrant Hoxb8 expression in implicated brain regions underlying OCD (206). The SAPAP3-knockout mice display excessive self-grooming starting at 4–6 months of age that leads to head skin lesions (178) and have dysfunction in cortico-striato-thalamo-cortico (CSTC) circuit (23, 29, 48, 99, 179, 180) components (207–211). SAPAP3 polymorphisms may be associated with early onset OCD (212) and grooming disorders (213) and SAPAP3 heterozygous variants are present in 4.2% of trichotillomania/OCD patients and in 1.1% of controls (137). Compulsive-like behavior in 5-HT2C knockout mice includes highly organized patterns of non-food chewing (137, 214), but they also have other abnormalities (214, 215). Slitrk5-knockout mice display compulsive-like grooming and cortico-striatal dysregulation (216) and rare mutations in this gene may be associated with OCD (138). Overexpression of the Slc1a1/EAAT3 glutamate transporter increases anxiety-like and repetitive behaviors in mice along with cortico-striatal deficits that is decreased to normal with the treatment of fluoxetine or clomipramine (217). This gene affects components of the CSTC circuit (130, 205) and Slc1a1/EAAT3 single nucleotide polymorphism have been associated with susceptibility for OCD (130, 218). These genetic models provide critical insight on the neurobiological aspects underlying pathophysiology of OCD.

## Face, Predictive, and Construct Validity of Animal Models for OCD

To mimic a human psychiatric condition, animal behaviors should have face, predictive and construct validity (219–221). Although OCD and other OCRD symptoms generally impair normal functioning (12–19), OCD-like behaviors in animals do not necessarily result in overt dysfunction, but are nevertheless very useful to mimic compulsivity common to OCD (219–221).

### Face Validity

Face validity refers to the degree of phenomenological similarity between the animal and the disease it is expected to model (201, 221). Phenomenological identity comprises of behavioral and or cognitive dimensions that mimic the disease symptomology (221). Considering the species divergence, the “extent of similarity” patterns are typically considered as qualifying criteria for an animal model of a disorder. Obsessions cannot be modeled in animals due to the specificity of the social and environmental contexts in humans that drive dimensions of obsessive thoughts (150, 201). Behaviorally, only the compulsions are manifested and therefore can be observed in animal models. Hence when cataloging OCD, behavioral patterns in existing animal models refer to the expression of an otherwise normal phenotype in an excessive and repetitive manner (149, 199, 204). The persistent and exaggerated behavioral patterns can be linked to the clinical dimensions of compulsivity as commonly observed in patient populations. Stereotypic grooming is the most commonly observed dysregulated innate behavior in animal models of compulsivity (201). Other spontaneous compulsive behaviors include patterned jumping, circling, backflips and summersaulting as seen in the deer mice model (222). Since the current review focusses on the spontaneous mouse model (140–145), we will restrict the review on the compulsive behaviors pertaining to the model that can serve as an useful tool to understand factors that modulate compulsivity in OCD and OCRD.

### Predictive Validity

Predictive validity relies on the therapeutic evaluation of an animal model to assess if first line therapy in the model in question mimics clinical potency (221). Predictive validity is not aimed at ensuring replication of translational specificity with respect to human pathophysiology but rather emphasizes on the therapeutic responses (221). A comparable response to the first line treatment options corresponds to a human-animal correlation of therapeutic outcomes. Predictive validity pertaining to OCD can only be limited to pharmacological or neurosurgical interventions since behavioral therapies such as cognitive behavioral therapy cannot be evaluated in animals (201, 223). To this end most animal models fulfilling predictive validity for OCD are evaluated for chronic regimens of selective serotonin reuptake inhibitors (SSRI), a class of drugs that show remission in certain patient subgroups (224). Additionally, neurosurgical methods such as deep brain stimulation has also been assessed in the SAPAP3-knockdown model of OCD (211). The SSRI typically function by blocking the serotonin transporters (SERT) thereby prolonging the serotonin concentration at the synapses for rewiring the neural substrates underlying behavioral anomalies (225, 226).

### Construct Validity

Construct validity refers to the comparable molecular and physiological mechanisms between the animal model and the disease it represents (221). Since the exact neural mechanisms of OCD are yet to be elucidated, construct validity of a model is reliant on correlative evidence from functional imaging studies on human patients (201). This typically involves substrates in the CSTC (23, 29, 48, 99, 179, 180, 227). Ovarian hormones, or the implicated neurotransmitter systems that can explain the behavioral correlates (face validity) and treatment response (predictive validity) are others (175). An alternative way to evaluate construct validity is to have animal models that recapitulate certain aspects of cognitive anomalies such as cognitive deficits, and reversal learning commonly observed in OCD patients (228, 229).

## Bidirectional Selection for Spontaneous Compulsive-Like Behaviors

The spontaneous compulsive-like mouse model was developed through bidirectional selection for high and low levels of nest-building from an HS/ibg outbred mouse stock population (181, 182, 230). The HS/ibg strain was derived through crossing of eight house mouse (Mus musculus) inbred strains [A, AKR, BLB/c, C3H/2, C57BL, DBA/2, Is/Bi, RIII; (181, 231)]. Bidirectional selection resulted in three levels of nest-building behavior (with two replicate strains within each level). The replicates within each level of nest-building were maintained as separate strains, i.e., not interbred with the other replicate strains, but subjected to the same selection regime (181, 182). This resulted in two HA strains (HA1 and HA3) that consistently display high and excessive levels of nesting with a 40-fold difference in the amount of cotton used when compared to the two LA strains (LA1 and LA2) which display very low levels of nesting. The LA strains are therefore considered non-compulsive. The two randomly-bred strains (CA1 and CA3) serve as a selection control and show intermediate levels of nesting (143, 182).

## Excessive Nest-Building and Marble Burying Fulfill the Validities for Compulsivity in Humans

As a naturalistic model, the spontaneously compulsive-like mouse strains exhibit two topographies of excessiveness and repetitiveness. They are compulsive-like nest builders (puling excessive amounts of cotton into their cage) and diggers (excessive burying of marbles) (140, 141, 143).

### Nest Building

For small rodents such as mice, nesting is a physiological adaptation that is critical for heat conservation, reproduction and to thwart predators (232–236). Healthy nesting is an essential indicator of welfare among animals and alteration in normal nesting patterns could be indicative of change in health or welfare (237). As nest-building is energetically costly, it can make rodents vulnerable to predation (238, 239). Hence it becomes imperative from an adaptation standpoint to create a dynamic balance between behavioral engagement and energy conservation (240). According to the security motivation theory of OCD, individuals engage in species-specific behaviors to handle potential danger and no consummatory stimuli should interfere with the impulse control of behavioral continuity (241, 242). Cotton acts as a consummatory stimuli for the compulsive-like strains that exhibit almost negligible latency to initiate nest-building when introduced to cotton for the first time showing rapid, forceful and repeated movement of the front paws and mouth for extended periods of time (140, 141, 143). Since latency to nesting is considered as a good indicator for motivation (240) such excessive and repetitive patterns of nest-building fails to prioritize conserved behavioral patterns mimicking loss of impulse control which is often reported in human OCD patients (243–245). This perseverant action leads to pulling of excessive amount of cotton through the cage top metal bars over prolonged periods of time leading to uncontrolled hoarding of nesting material (140, 141, 143). Both the sexes of the HA strains accumulate massive amounts of cotton in the cages that are incorporated into a functional nest and in most animals, the cotton fills the entire cage. This is in stark contrast to the normal nesting phenomenon in the control strains (CA strains) which appears to be focused on energy conservation and the building of a functional nest that typically have the minimal amount of nesting material required (140, 141, 143). The small nest-builders (LA strains) do not collect sufficient amounts of cotton to build a functional nest (181, 182, 234). Therefore, nest-building in the big nest-builders (HA strains) recapitulates several dimensions of compulsive behaviors with repetitiveness and excessiveness that shows face validity for understanding OCD.

With greater knowledge of the neurobiological underpinnings mediating nesting behavior it is evident that learning and memory play a significant role in expression of this phenotype. The hippocampus and prefrontal cortex are brain regions typically implicated in nest-building. Studies have indicated that lesions in the hippocampus or PFC disrupt nest formation (246–249). Further, disrupting GABAergic signaling in the neuropeptide Y interneurons of the PFC augments nest-building phenotype (250). Similarly, perturbing serotonin signaling results in poor nest construction (251). Nest-building also integrates intricate sequences of discrete motor learning that involves alterations in physical actions often in a stereotypic manner. Such perseverant action parallels reward based training paradigms indicating involvement of brain substrates underlying motor and motivational behaviors (252). Dopaminergic activation in reward circuitry is one of the molecular hallmarks of nesting behavior which integrates incentive and reinforcing phenotypes (252). Nest-building therefore encompasses sensorimotor, goal-directed and reward based neural mechanisms involving several neurotransmitter systems. Since, aberration of the abovementioned regions and neurotransmitter mechanisms has been documented in several studies investigating clinical populations (253–259), nest-building can be considered as a relevant and suitable behavioral phenotype to address some aspects of compulsivity in humans.

### Marble Burying

Burying and digging are core behavioral attributes in rodents (260). These behavioral repertoires often involve stereotypic actions that align to more specific outcomes. For example, burying allows displacement of noxious or non-noxious objects while digging involves displacement of substrates (261). Marble burying behavior is one of the most widely studied models to assess compulsive-like phenotypes (140, 143, 262–265). The concept of marble burying utilizes the natural tendency of mice or rats to bury harmless or noxious objects in the bedding provided to them (174). Since studies (266–268) have shown that rodents typically do not tend to avoid the marbles, or get habituated after repeated exposures, marble burying is a good indicator of a compulsive-like but not an anxiety-like phenotype (140, 267, 268). The compulsive theory of marble burying is based on the fact that non-reactivity of the marbles fails to provide stimuli for ending the investigation leading to frustrated burying of objects that gets stereotypic in nature (149). Marble burying can be mapped to the inability to realize task completion in OCD patients (241), thereby establishing its face validity. Similar to nest-building, marble burying requires hippocampal and cortical regions for functional outcomes (260, 269). Marble burying also integrates motor regions such as the striatal components for executing repetitive locomotor actions mediating the compulsive digging behavior (267, 268, 270, 271). Further, several neurotransmitter systems have been implicated in influencing the behavior (272–276). The compulsive-like HA strains demonstrate a persistent compulsive-like marble burying behavior (140, 143). Most mouse models tested for marble burying as an indicator of compulsive-like behavior typically bury on an average 10–12 marbles (222, 277, 278). It is interesting to note that such burying attributes often correlate to anxiety-like and not compulsive-like responses. On the contrary, compulsive burying in the HA strains comprise of aggravated and stereotypic digging of the marbles averaging 18–20 marbles (140–145, 279). Upon completion of digging, mice often appear agitated with rapid locomotor movements and perform frenzy investigation of the apparatus. Further, the HA strains engage in burying behavior throughout the entire duration of the test despite marbles being buried. The non-compulsive-like LA strains on the other hand bury 6–8 marbles on an average which is often accompanied by freezing behavior and lower locomotor activities, while the CA control strains bury 10–13 marbles with intermediate motoric activity (140, 141, 143). The non-compulsive-like LA strains therefore conform to a more anxiety-like phenotype that can be characterized as risk aversion (280, 281), while the control strains lie intermediate on the spectrum.

This abnormal behavioral excessiveness exhibited through nest-building and marble burying in the spontaneous compulsive-like mouse strains qualifies these mice to be a suitable naturalistic model to investigate behavior and the nervous system by proxy. The presence of non-compulsive-like LA and control strains further strengthens the phenotypic rigor in terms of establishing a behavioral gradient across strains to better understand the behavioral anomaly expressed as a function of genetic selection and variation. Further, nest-building and marble burying behaviors are malleable to genetic (181, 182), pharmacological (140, 142, 145, 279) and environmental (181, 182) manipulations serving as important tools for dissecting neural substrates mediating functional outcomes. Nest-building behavior draws a parallel with hoarding in human patients as reported earlier, while marble burying can recapitulate failure of task completion that falls in the realm of compulsive checking as seen in many OCD patient subsets (241). Excessive nest-building and marble burying in the compulsive-like HA mouse strains can also form disparate compulsive-like traits (145) that can be utilized to better understand disparate compulsive traits in patient subgroups. Moreover, the degree of nest-building in the HA strains always exhibits consistent and positive correlation with marble burying providing a basis for comparison of behavioral traits through sex, treatment and physiological differences (140–145, 279).

## Adjunct Behaviors in the Spontaneous Compulsive-Like Mouse Model

Apart from expressing variable compulsive-like behaviors, the nest-building mouse strains exhibit an array of other adjunct phenotypes that vary among the three nesting phenotypes and between replicates within each phenotypes which is also influenced by sex differences (143). Such variable expressions pertain to anxiety-, depression- and cognitive-like domains (143). Comorbidities are a critical component of the symptomatic repertoires in OCD patients. These comorbid symptoms vary within clinical subgroups and often make prognosis cumbersome (88). Most animal studies so far have focused on dissecting compulsive-like behaviors as a function of behavioral, genetic, pharmacological and circuit-based manipulations (see sections Spontaneous Animal Model for OCD, Induced Compulsive-Like Behaviors, and Genetically Modified Mice That Express Compulsive-Like Behaviors above). Characterization of associated phenotypes have been largely unexplored in various model organisms for OCD. We have exhaustively identified the domain specific behavioral expression profile of the nesting mouse strains. In general, the compulsive-like HA strains show less anxiety on anxiety-like assessments in open field and elevated plus and zero mazes (140, 141, 143). Interestingly, anxiety-like responses in the compulsive-like mice vary based on sex, strain differences and on the difficulty level of the tests allowing to identify distinct behavioral patterns across variable emotional dimension of affective behaviors. For example, the male HA strains exhibit strain differences when (141) evaluated on elevated plus maze but show no such replicate strain variation when tested for the open field (143). Moreover, HA2 females and HA1 males show a negative correlation between specific compulsive-like traits and anxiety-like responses. Nest-building in the HA2 females and marble burying in the HA1 males correlate with increased anxiety in the elevated plus maze indicating a complex genetic architecture driving functional outcomes that parallel clinical heterogeneity (143). The non-compulsive-like LA strains on the other hand exhibit anxiogenic responses in the anxiety-like evaluations of open field and elevated mazes (140, 143). For cognitive-like tests as determined through novel-object recognition, one of the CA female strains has higher object recognition memory when compared to the respective males. No differences are observed for the HA and the LA strains. For depression-like forced swim behavior, the strains display a significant sex and sex by strain interaction effects (143). At the biochemical level, the compulsive-like HA strains and the non-compulsive-like LA strains mount higher corticosterone levels than the selection controls (143). These variations could be attributed to genetic drift or founder effects (181, 182). Such complex behavioral expression could be essential in understanding the symptomatic framework and underlying neural substrates of OCD subgroups. Several heritability studies have pinpointed toward a lack of single identifier gene and often report an interaction of multiple gene networks that overlaps with environmental factors resulting in discrete expression of primary symptoms and associated comorbidities (29). Thus, it becomes imperative to examine an animal model that displays both primary and secondary behavioral attributes that is structured upon genetic variations due to bidirectional selection.

## Strain and Sex Differences in Spontaneous Mouse Model: Recapitulating Clinical Heterogeneity

OCD most likely has a polygenic basis. Family studies, segregation analysis studies, candidate gene studies and genome wide association studies have consistently reported and established heritability and genetic mechanisms as important causative factors of OCD. Family studies have indicated that early onset OCD has a familial basis (282, 283). Family studies with adult probands have revealed that OCD is familial with increased risk of occurrence among relatives of patients with childhood onset OCD (284–286). Around 100 candidate gene studies have focused on the genetic variants predominantly within neurotransmitter pathways (127). One of these candidate gene studies has shown that OCD might be associated with polymorphisms in serotonin system related genes such as 5HTTLPR (serotonin-transporter linked polymorphic region) and HTR2A (5-hydroxytryptophan receptor 2A) (287). The same study also pinpointed genetic variations in monoamine oxidase and catechol-o methyl transferase genes only in male patients and dopamine system related genes such as DAT-1 (Dopamine active transporter 1 gene) and DRD3 (Dopamine receptor D3) (287). In addition, other genome wide association studies have identified various genes that code for essential neurobiological substrates (53, 288–290), which are critical for neuronal transmission and plasticity, such as DLGAP1 (guanylate kinase-associated protein) and BTBD3 (BTB domain containing protein 3). Despite such exhaustive attempts, these GWAS studies have yielded unreliable results with no specific hits and minimal replication of the aforementioned genes that can be conclusively tied to the pathophysiology of the disorder. Interestingly, a recent study identified large-effect size causes pertaining to *de novo* coding variants as risk factors for OCD (291).

Earlier onset of OCD in males when compared to females (292), bimodal distribution of onset in females and exacerbation of symptoms during various reproductive phases (293) point toward the role of steroid hormones in OCD. Sex hormones are mainly steroids that are produced by the gonads (294). The most relevant are female sex hormones estrogen and progesterone, and the male sex hormone testosterone. These hormones regulate and influence global sexual functions like pregnancy, pubertal changes and sexual behavior (295). Estrogen and progesterone have neuromodulatory effects in the central nervous system either through intracellular action or *via* neurotransmitter systems (296–298). These sex steroids further metabolize into neurosteroids, which have anti-convulsive (299), anti-depressant (300) and anti-anxiety (301) properties through modulation of GABA and glutamate receptors (302). The emphasis of sex steroids in influencing onset and exacerbation of OCD symptoms are more relevant to female OCD patients. This is mainly because women are at risk of developing the disorder during various reproductive phases (303).

Nesting can be attributed to polygenic mechanisms and often exhibit a complex genetic architecture (181, 182). There are several genomic regions that are associated with the behavior and often exert dominance and additive effects (181, 304–306). Realized heritability for nest-building is 0.30 (181, 182). This is in the same range as heritability estimates for OCD that range from 26 to 65%, which may depend on symptom dimension (29, 39, 104–106). Replicate diffferences within the same nesting phenotype, i.e., HA, control, and LA strains, for other behaviors and traits are most likely due to founder effects and/or genetic drift (181, 182). Therefore, comparing nest-building phenotype and other behaviors and traits across replicate strains and nesting phenotypes can be important to understand how polygenic mechanisms contribute to variation in compulsive expression and the implicated neural framework. Further, such strain comparisons can form the basis for dissecting how compulsivity diverges based on individual genetic variation largely contributing to clinical heterogeneity. Strain comparisons also allow drug response variations that can form the basis to examine differential treatment response rates often seen across patient subgroups. Such evaluations will enable a holistic approach toward understanding the pathophysiology. To this end we have conducted studies that have unveiled substantial understanding of how strain differences contribute to divergent phenotypic and drug response profiles in the compulsive condition.

For compulsive-like behaviors, the HA strains respond only to first line treatment options (140). There is dose-dependent attenuation of compulsive-like nesting and marble burying behaviors in the HA males treated with chronic fluoxetine and clomipramine (140), and fluvoxamine (145). However, desipramine, a tricyclic antidepressant which is not effective in treating OCD (307–310), also shows no significant effects on compulsive-like nesting and marble burying (140) indicating specificity and predictive validity of first line treatments for attenuating the compulsive-like phenotypes. Using an alternative first line drug results in differential behavioral outcomes pertaining to the compulsive-like domains. On being subjected to chronic regimen (2 weeks) of fluvoxamine a dose-dependent attenuation of nest-building is observed only in the HA1 but not in the HA3 males (145). For compulsive-like marble burying trait, fluvoxamine shows comparable results with overall dose-dependent decreases in marbles burying in both the strains. Currently there is no consensus on the best treatment option for OCD (30). Relapse rates with SSRIs are high in clinical populations (311). Medication differences have also been observed with these first-line treatments such as in children with OCD and comorbid tics, which reduces the efficacy of SSRI treatment (312). It is however unclear if the same effect is persistent in adults (313). Among various factors, compulsive traits and associated co-morbid symptoms can result in non-responsiveness to treatments in the OCD condition (88, 314). Hence, a trait and dosage specific response to first-line therapies in the HA strains cannot be ruled out.

To better understand the role of strain and sex differences we have demonstrated that the HA strains exhibit strain and sex differences in expression of behaviors (143). When tested in the proestrus stage of the estrus cycle when the estrogen levels are thought to be higher in circulation, the HA1 and HA3 females exhibit less compulsive-like nest-building than the males, exhibiting a sex difference in expression of compulsivity. This sex difference is however retained only in the HA1 strains for marble burying behavior (143). The HA strains exhibit no strain differences in the open field. However, in the elevated plus maze the HA3 males explore the open arms more than the HA1 males while the female HA strains exhibit no such differences (143). Further, open arm explorations of both the HA females and HA1 males in the plus maze follow a negative correlation with the compulsive-like nest-building and marble burying behaviors (143). Such behavioral expression patterns are critical in delineating the different aspects of emotionality associated with anxiety that could be driven by sex and genotype interactions in the compulsive condition.

## Role of Physiological State in Modulating Compulsivity

There is limited understanding as to how the physiological status that is largely driven by hormonal fluctuations influence OCD pathophysiology, which is especially relevant in women. With greater susceptibility of females for certain psychiatric disorders limited investigations have been conducted with contradictory results (315). One study has shown more prevalence of OCD during menopause (316), while another has demonstrated that the symptoms are more related to menarche and decrease during menopause (317). Acute conditions, such as surgical menopause, is more abrupt and can mimic natural menopause (318) leading to more drastic depletion of ovarian hormones precipitating mood and anxiety disorders (318). Interestingly, anti-compulsive effects of estradiol, allopregnanolone and progesterone have been established in animal studies (148, 263, 319, 320). When subjected to acute ovariectomy, exacerbation of compulsive-like and anxiety-like behaviors are observed in the compulsive-like female mice, but not in the CA control and LA strains, with a trait specific variation in compulsive-like behavior among the two female HA strains (141). This inter strain effect is abolished in the compulsive-like nest-building phenotype but not for marble burying once the ovariectomized strains were treated with either 17β-estradiol (E2) or progesterone (P4). Acute E2 administration attenuates compulsive-like nesting and marble burying, while P4 administration show no effect on these behaviors (141). For the anxiety-like measures a strain specific response to E2 and P4 administration is observed (141). Overall these behavioral attributes adhere to a complex heterogeneity (321–323) and reveals the interaction of genetic variation and sex hormones.

The post-partum condition is often associated with mood fluctuations and psychopathology and can precipitate OCD symptoms (324, 325). Physiologically, the brain undergoes dramatic shifts in hormonal milieu. This is often characterized by activation of oxytocinergic system in the paraventricular nucleus (PVN) and supraoptic nucleus (SON) regions of the hypothalamus due to suckling action (326–328). This leads to secretion of prolactin in the duct system. Since, both oxytocin and prolactin have functional roles in affective behaviors (329, 330), hindering the lactation process can alter cellular plasticity leading to mood disorders. This disruption can be further aggravated in patients with existing mental conditions such as OCD (331). However, the influence of post-partum condition on OCD symptomology is not known. Further, the neurobiological mechanisms underlying the interaction of post-partum phase and compulsivity is also unexplored. Using the HA1 strain we have also furthered the understanding of the role of oxytocin in influencing compulsive-, anxiety- and depression-like behaviors in post-partum condition. We have shown that oxytocin due to lactation is anti-compulsive during the post-partum phase and blocking oxytocin receptor exacerbate compulsive-like nest-building and marble burying behaviors during lactation (144). This behavioral modulation by oxytocin correlate with altered serotonin immunoreactivity in the dorsal raphe nucleus and enhanced responsiveness to fluoxetine. This indicates cross-talk between the oxytocin and the serotonergic signaling pathways during the compulsive-like condition and shows that suckling action may be beneficial during the post-partum condition (144). These findings provide critical insight into the putative role of oxytocin during physiologically challenging conditions such as post-partum phase warranting further investigation on the role of SSRI effectiveness.

## Effect of New Pharmacotherapies and Psychostimulants in the Spontaneous Mouse Model

There is evidence of cholinergic involvement in OCD (332, 333). OCD patients typically report less smoking behavior when compared to other neuropsychiatric disorders (334). It has been hypothesized that nicotine further activates the hyperactive fronto-striatal circuit resulting in exacerbation of symptoms. This is mainly through glutamate release by nicotinic receptor activation (334). This theory has however been challenged by other studies which have shown significant clinical improvements in OCD patients augmented with nicotine (335). Since cholinergic projections innervate the orbitofrontal cortex (336), which is one of the implicated regions in OCD (229, 337), further investigation is required to understand the modulatory role of cholinergic system in obsessive-compulsive behaviors. It was not until recently that cholinergic involvement was investigated in animal models of OCD. Selective ablation of striatal cholinergic interneurons results in repetitive behaviors concomitant with ritualistic social exploration in a transgenic mouse model (338). Congruently, we recently demonstrated that positive allosteric modulation of nicotinic receptor α4β2 subtype by a novel drug, desformylflustrabromine (dFBr), attenuates compulsive-like but not anxiety-like behaviors in the compulsive-like mouse model (142). The anti-compulsive dose-dependent effect of dFBr is observed in both acute and chronic administration regimens (142) indicating that targeting the α4β2 nicotinic receptor subtype could result in rapid remission of compulsivity and is a potential therapeutic area that requires attention.

Psychostimulants influence the pathophysiology of psychiatric disorders. With a surge in the unregulated usage of psychostimulants (339), animal models of psychiatric disorders should be investigated for their potential modulatory effects on behavioral expression. Considering the clinical heterogeneity among patient populations as discussed, it is feasible that psychostimulants will have differential effects among various patient subgroups. Caffeine is the most commonly used psychostimulant consumed by over 90% of the adult population and have several health enhancing effects (340). However, caffeine also causes cardiovascular disorders, sleep disturbances, and has an abuse liability (341). The specific role of caffeine in influencing obsessions and compulsions remains to be determined. In fact, OCD patients with comorbid bipolar disorders become easily addicted to caffeine indicating possible interactions between caffeine and OCD traits (342–345). Acute exposure to a high dose of caffeine reduces both nest-building and marble burying behavior in the compulsive-like HA strains (279). On the contrary, a chronic exposure of a high dose of caffeine increases nest-building behavior without influencing marble burying indicating trait and drug response heterogeneity (279).

## Conclusion

One of the greatest challenges in OCD research has been to pinpoint neurobiological substrates and devise effective therapeutic measures targeting the same. Clinical heterogeneity (346) further compounds the problem resulting in higher drug resistance rates (37). Animal models to this end have been resourceful for identifying candidate genes that account for behavioral anomalies (203) and drugs of therapeutic value. However, much of the pre-clinical knowledge has not been successfully translated to the clinic. It is important to acknowledge that OCD is caused by a conglomeration of various factors (29). Hence, it becomes essential to account for those variables while selecting and testing an animal model. For example, strain comparisons can be critical in understanding heterogeneity in behavioral expression and drug responses that correlate to compulsive responses in humans. Rodent models such as the spontaneous compulsive-like nest-building mice exhibit variation in intensity of compulsive-like phenotypes. Many studies on animal models of OCD do not consider females when testing for face, predictive and construct validity. Sex differences play a vital role in the disease dimensions pertaining to onset, symptom patterns and associated comorbidities thereby accounting partially, if not completely, toward clinical heterogeneity. Further, physiologically challenging conditions such as puberty, pregnancy, post-partum and menopause modulate OCD. Future studies on both animals and humans should therefore consider sex differences and take into consideration the physiological stages for better evaluation of these factors in disease pathophysiology.

The spontaneous compulsive-like mice (HA strains) exhibit consistent, predictable and reproducible compulsive-like phenotypes across both sexes and provide a basis for investigating variables that might influence disease pathophysiology. Due to the established role of environmental influence in OCD, specific triggers such as prenatal infections, trauma and stress should be incorporated while subjecting animals to behavioral tests. For example, a multitude of studies have indicated that maternal immune activation result in exacerbation of compulsive-like marble burying behavior in offspring (347–349). Further, inflammation interacts with genetic factors to potentiate behavioral impairments (350). Greater precedence is therefore needed to devise such model systems that look at gene-environment interactions in compulsive-like expression. The spontaneous compulsive-like mouse strains (HA strains), which were developed through bidirectional selection for nesting (181, 182), provide the unique opportunity to tap the interaction of such environmental and genetic factors (140–145) as we have reviewed here.

OCD is often associated with social impairments and has been consistently reported in patient cohorts (351). It could therefore imply that in certain patient subsets the brain circuitries pertaining to social interaction and compulsivity is affected. Animal models should be evaluated for such complex paradigms where two or more behavioral domains are tested simultaneously. One study with the naturalistic deer mouse model showed deficits in social interactions that correlated with compulsive-like phenotypic expression (352). In another study, clomipramine exposure to immature rats resulted in increased anxiety, inflexibility in alternation and reversal learning, working memory deficit and hoarding (353). Similar exhaustive investigation with the spontaneous compulsive-like mouse model on multiple behavioral domains can reveal overlapping brain regions effected in OCD.

It is also critical to look beyond the boundaries of the rudimentary behaviors that have been defined in the literature. Inch-worming, a novel pattern of digging, has been reported in BTBR mice that can recapitulate compulsive behaviors in humans (354). With persistent digging commonly observed across the HA strains, micro-behaviors within a general behavioral domain can elucidate several aspects of repetitiveness. Time spent digging and patterned digging could provide a better representation of the behavioral attributes mediating compulsivity. Certain subsets of the compulsive-like HA mice also exhibit compulsive-like food shredding phenotype that is repetitive in nature (unpublished data). Such food grinding/shredding in captivity has been suggested to represent compulsive-like behavior (355, 356) and varies considerably based on environmental factors such as diet and enrichments. Food shredding is observed in a subset of mice of the compulsive-like strains and the role of genetics, such as founder effects and genetic drift (181, 182), cannot be ruled out. Hence food shredding can be utilized as a novel compulsive-like phenotype to understand the role of gene-environmental interactions.

Like every other model of compulsive-like behaviors the spontaneous mouse strains also have some limitations. The compulsive-like HA strains do not exhibit pathological grooming behavior, a phenotype common to several spontaneous, gene knockout and drug-induced OCD models. Moreover, the putative polygenic factors driving such transgenerational compulsive-like phenotypes are unknown. This however does not take away the importance of these strains in providing critical insight into the heterogeneity of repetitive behaviors and differential drug responses that are modulated by gene, environment and sex interactions. Further, identifying genes regulating these repetitive nesting and burying behaviors through modern genomic approaches can divulge new targets previously unidentified and linked to the pathophysiology of OCD.

Finally, we have demonstrated in this review that artificial bidirectional selection for a normal behavior, i.e., nest-building behavior, resulted in the creation of a spontaneous, stable and predictable compulsive-like phenotype that has face, predictive, and construct validity for studying various aspects of OCD in humans. Specifically, construct validity (201) has been established by desipramine having no effect on compulsive-like nest-building behavior (140) and the involvement of the serotonergic (140, 145, 357), cholinergic (142), estrogenic (141), oxytocinergic (144), and GABAergic neurotransmitter pathways (357). In addition, the use of these selected strains resulted in the identification of an entirely new class of drugs for attenuating compulsivity, i.e., drugs that affect the nicotinic receptor α4β2 subtype as shown with dFBr, a positive allosteric modulator of these receptors (142). Importantly, dFBr is effective within 2 h and reduces compulsive-like behaviors up to 90% (142). Therefore, the use of artificially selected mouse strains for compulsive-like behaviors represent an alternative strategy to study OCD and OCRD that has great potential.

## Author Contributions

SM wrote the first draft of the manuscript. AB-I made extensive revisions of the manuscript. All authors contributed to the article and approved the submitted version.

## Funding

The research reported in this publication on the spontaneous, stable and predictable compulsive-like behavioral phenotype was funded by grants from the National Institute of General Medical Sciences of the National Institutes of Health (1) under an Institutional Development Award (IDeA; Grant Number P20GM103395) and (2) under a Building Infrastructure Leading to Diversity Award (BUILD; three linked Grants Numbered RL5GM118990, TL4GM118992, and 1UL1GM118991). The Department of Biology and Wildlife and the Department of Chemistry and Biochemistry at the University of Alaska Fairbanks also supported this work.

## Author Disclaimer

The work is solely the responsibility of the authors and does not necessarily represent the official view of the National Institutes of Health.

## Conflict of Interest

AB-I is the President and CEO of OCRD Biomed LLC, which is pursuing patents for the use desformylflustrabromine as a novel treatment for obsessive-compulsive and related disorders. The remaining author declares that the research was conducted in the absence of any commercial or financial relationships that could be construed as a potential conflict of interest.

## Publisher's Note

All claims expressed in this article are solely those of the authors and do not necessarily represent those of their affiliated organizations, or those of the publisher, the editors and the reviewers. Any product that may be evaluated in this article, or claim that may be made by its manufacturer, is not guaranteed or endorsed by the publisher.
